# Burden of illness of hip fractures in elderly Dutch patients

**DOI:** 10.1007/s11657-019-0678-y

**Published:** 2020-01-03

**Authors:** T. A. Kanters, C. L. P. van de Ree, M. A. C. de Jongh, T. Gosens, L. Hakkaart-van Roijen

**Affiliations:** 1 Institute for Medical Technology Assessment, Erasmus University Rotterdam, Burgemeester Oudlaan 50, PO Box 1738, 3000 Rotterdam, DR Netherlands; 2 Department Trauma TopCare, Elisabeth-Tweesteden Hospital, Hilvarenbeekseweg 60, 5022 Tilburg, GC Netherlands; 3 Brabant Trauma Registry, Network Emergency Care Brabant, Elisabeth-Tweesteden Hospital, Hilvarenbeekseweg 60, 5022 Tilburg, GC Netherlands; 4 Department of Orthopaedic Surgery, Elisabeth-Tweesteden Hospital, Hilvarenbeekseweg 60, 5022 Tilburg, GC Netherlands; 5 Health Technology Assessment, Erasmus School of Health Policy and Management, Erasmus University Rotterdam, Burgemeester Oudlaan 50, PO Box 1738, 3000 Rotterdam, DR Netherlands

**Keywords:** Hip fractures, Elderly, Burden of illness, Costs, Health-related quality of life

## Abstract

***Summary*:**

Patients with hip fractures experience reduced health-related quality of life and have a reduced life expectancy. Patients’ utilization of healthcare leads to costs to society. The results of the study can be used in future economic evaluations of treatments for hip fractures.

**Purpose:**

Hip fractures are associated with high mortality, reduced quality of life, and increased healthcare utilization, leading to an economic burden to society. The purpose of this study is to determine the burden of illness of hip fractures in elderly Dutch patients for specific time periods after surgery.

**Methods:**

Patients with a hip fracture above the age of 65 were included in the study. In the 1-year period after surgery, patients were asked to complete a set of questionnaires pre-injury (retrospectively), and 1 week, 1 month, 3 months, 6 months and 12 months after surgery. The set of questionnaires included the Euroqol 5D (EQ-5D-3L), the iMTA Medical Consumption Questionnaire (iMCQ) and iMTA Productivity Cost Questionnaire (iPCQ). Health-related quality of life was calculated using Dutch tariffs. Costs were calculated using the methodology described in the Dutch costing manual.

**Results:**

Approximately 20% of patients with a hip fracture died within 1 year. Health-related quality of life was significantly reduced compared to pre-injury values, and patients did not recover to their pre-injury values within 1 year. Total costs in the first year after injury were €27,573, of which 10% were due to costs of the procedure (€2706). Total follow-up costs (€24,876) were predominantly consisting of healthcare costs. Monthly costs decreased over time.

**Conclusions:**

Hip fractures lead to a burden to patients, resulting from mortality and health-related quality of life reductions, and to society, due to (healthcare) costs. The results of this study can be used in future economic evaluations.

**Electronic supplementary material:**

The online version of this article (10.1007/s11657-019-0678-y) contains supplementary material, which is available to authorized users.

## Introduction

Hip fracture is a severe fracture attributable to bone fragility and predominantly affects an already frail population. A distinction can be made between low-energetic and high-energetic traumas, with low-energetic traumas affecting an older population. The burden of hip fractures on healthcare and society is very high. In 2010, the number of hip fractures in the European Union was over 600,000 [1]. Given the ageing population, the number of hip fractures is projected to grow in the coming decades [2]. Hip fractures can result in complications, chronic pain, reduced quality of life and premature death [3–5]. Next to the clinical burden to patients, hip fractures lead to medical consumption, including hospitalizations, and associated healthcare costs. In 2010, the estimated economic burden of hip fractures in the European Union due to use of healthcare services was €19 billion [1]. In addition to the burden on the healthcare budget, a patient’s social environment is likely to be affected, because of emotional reasons and because of an increased need for informal care [6]. Finally, hip fractures potentially result in productivity losses, particularly due to a patient’s inability to perform unpaid work, as the proportion of patients with paid work is generally small due to the population’s high age. The prevention and optimal treatment of hip fractures are therefore of crucial importance. The efficiency of prevention and treatment options is, given the economic burden, likely to be an increasingly important factor deciding on the care pathway. For this purpose, data on both cost and effects are required to inform health economic models. One of the benefits of using models for economic evaluations is their ability to extrapolate outcomes beyond the observed period. For this purpose, it is crucial that the pattern of costs and health-related quality of life (HRQOL) over time is identified, because costs and HRQOL can be different directly after surgery than after a period of time.

Previous publications on per patient healthcare costs of hip fractures in the Netherlands have reported cost estimates between €19,741 and €26,355 (inflation corrected to 2018 values) [7–10]. Next to costs, information on HRQOL is crucial for health economic studies. HRQOL is generally presented in a utility value, which scores HRQOL on a scale from 0 (death) to 1 (perfect health). The impact of hip fractures on HRQOL has previously been assessed in the Netherlands in other studies. A study in the period 2001–2002 showed that utility scores for patients with hip fractures were severely reduced compared to the average Dutch population: 2.5 months after injury, the average utility was 0.43 and 0.67 after 24 months [11]. More recent Dutch studies did not report utility values but have been reported in international studies. A recent systematic review showed that HRQOL deteriorates in the first period after a hip fracture, after which patients recover to a level below their pre-injury level [5]. The primary objective of the current study is to provide a comprehensive overview of the burden of illness of hip fractures in an elderly population in the Netherlands. For this purpose, we examine, over a period of 1 year after hip fracture, life expectancy, HRQOL and healthcare and productivity costs in a sample of Dutch elderly patients with a hip fracture who underwent surgery. A distinction in monthly costs and utilities over time will be examined, so that these estimates can be used in model-based economic evaluations.

## Methods

### Dataset

The study used the Brabant Injury Outcome Surveillance (BIOS) database, a multicentre observational follow-up cohort study, which is described elsewhere [12]. Data were collected in the period August 2015–November 2016. For the current study, the subset of hip fracture patients was selected. The dataset contained 821 patients with hip fracture over the age of 65 with a hip fracture that underwent surgery. Patients were followed for 1 year, with measurements of pre-injury (T0; measured retrospectively), and 1 week (T1), 1 month (T2), 3 months (T3), 6 months (T4) and 12 months (T5) after injury. The dataset contained information on survival, HRQOL, frailty and healthcare costs and productivity costs. Only patients who participated in the prospective study were included in the sample. Patients that died during hospitalization (*n* = 2) were excluded from the study, since they were unable to provide informed consent after surgery. Furthermore, three patients with pathological fractures were excluded. No patients were excluded because of language barrier, although language barrier was an exclusion criterion. Ethical approval was received from the Medical Ethics Committee Brabant, the Netherlands (NL50258.028.14). Informed consent was obtained from all individual participants included in the study.

### Measurement and valuation of health-related quality of life

HRQOL was expressed in utilities, derived from the EQ-5D-3L. This generic instrument is used to measure health status using five health dimensions (mobility, self-care, usual activities, pain/discomfort and anxiety/depression), each dimension having three levels [13]. Health status descriptions from the EQ-5D can be valued using tariffs from preference elicitation studies to calculate utilities, which can be used in economic evaluations. Utility scores were derived from the EQ-5D using the Dutch value set [14]. Negative values were also possible and represent health states worse than death. Utility values calculated with the Dutch value set range from − 0.329 to 1.000.

### Measurement and valuation of costs

Costs of the surgical procedure were estimated using a micro-costing study. For this purpose, the time duration of all hip surgery procedures in 2017 in level 1 trauma centre Elisabeth-Tweesteden Ziekenhuis (ETZ, Tilburg, the Netherlands) was used. The costs associated with using the operation room (per minute, including overhead costs) and costs of prostheses were based on information from the financial department of ETZ. Involvement of medical personnel was based on expert opinion and valued using the Dutch costing manual [15].

Medical consumption was measured with the iMTA Medical Consumption Questionnaire (iMCQ) [16]. The questionnaire included questions on utilization of home care, general practitioner, rehabilitation, long-term care, psychologist and paramedical care. Except for the initial hospitalization resultant of the hip fracture and one outpatient visit following surgery (based on expert opinion), hospital costs were not included in the database. Dutch health economic guidelines require studies to be carried out from a societal perspective, meaning that all costs and effects should be included in the analyses [17]. Therefore, not only healthcare costs were included in the study. The iMTA Productivity Costs Questionnaire (iPCQ) was used to measure productivity costs [16, 18]. Data on unpaid work (e.g. household activities) were not collected. Healthcare consumption and productivity losses were valued using the most recent update of the Dutch costing manual [15]. The friction cost method was used to establish productivity costs. Prices were indexed to 2018 price level. Data on informal care and associated costs were not available.

### Missing data

To make optimal use of available data, missing data were imputed. For this purpose, HRQOL was imputed using multiple imputation by chained equations [19] and linear interpolation. Costs were imputed using multiple imputation and mean imputation for individual items. Full details about the imputation procedures are provided in the appendix.

### Statistical analyses

Utility values were presented for patients alive at each specific time point. In addition to utility values, QALYs were calculated by combining survival and quality of life. Since the follow-up of the current study was 1 year and utilities are maximized at 1.00, the maximum QALY value in this study was 1.00. Costs were presented as total annual costs and average monthly costs for specific time periods. Total annual costs include cost estimates for patients that died during the study period.

Subgroup analyses were performed with respect to 1-year survival (whether patients survived the first year after injury or not), gender, age (age groups of 65–69, 70–79, 80–89 and ≥ 90), comorbidity, pre-injury living situation and frailty. Statistical analyses were performed using Stata 15.1 (StataCorp).

## Results

The average age in the patient population was 80 years (SD of 8.63; range 65–101). The majority of patients were female. More than 80% of patients had one or more comorbidities at moment of injury. Most common comorbidities were heart malfunctions (29% of patients), arthrosis (28%), dementia (23%) and osteoporosis (18%). Pre-injury HRQOL was 0.72 (SD of 0.28; range of − 0.204 to 1.00). Half of the patients were identified as frail on the Groningen Frailty Indicator (GFI). A total of 21% of patients lived in an institution pre-injury (Table 1.)

**Table 1:** Patient characteristics

Variable	Mean	Std. err.
Age	80.2	0.349
Gender (% female)	70.3%	0.018
One or more comorbidities pre-injury	82.5%	0.015
Health-related quality of life pre-injury	0.722	0.011
Frail elderly (GFI ≥ 4) pre-injury	52.1%	0.021
Living in an institution pre-injury	21.2%	0.016

GFI: Groningen Frailty Indicator [20]

### Survival and health-related quality of life

Survival data were available for 820 patients. The survival rate in the first 30 days after injury was 99.5% [95% CI: 98.7–99.8]. One-year survival was 83.3% [95% CI: 80.3–86.0]. Mortality of 80-year-olds in the Dutch population is 4.3% [21].

**Fig. 1:**
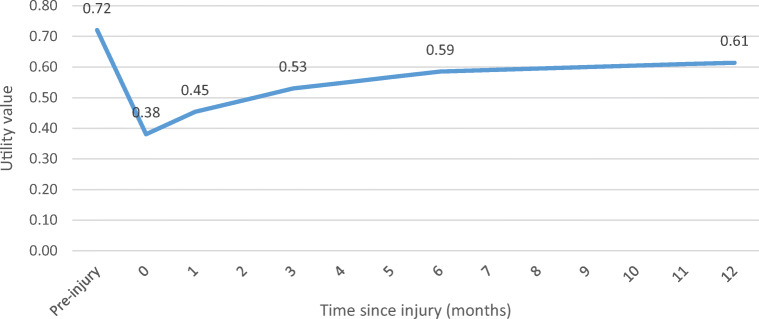
Utility values for patients with hip fracture over time

Pre-injury HRQOL data were available for 625 patients. Pre-injury HRQOL for this patient population was on average 0.72. This is approximately 13% lower than the Dutch population norm utility value of 0.83 of people over the age of 75 [22]. Figure 1 shows the development of utility values in the first year after injury. Hip fractures resulted in a sharp decrease in HRQOL compared to patients’ pre-injury utility value. With time, patients gradually recovered from the hip fracture, but their utility value after 1 year was still substantially lower than their pre-injury utility.

The average QALY value for patients in the year during follow-up was 0.528 (95% CI: 0.504–0.553).

### Costs

Table 2 shows that total costs following the first year after a hip fracture were €27,573. Cost of the surgical procedure was €2706 (range of €1734 – €4397), accounting for 10% of total costs. Data on costs in the period following surgery were available for 663 patients in the BIOS study. Table 2 shows that average total healthcare costs in the year following injury were €24,760 (range of €21,113–€28,406), accounting for 90% of total costs. Productivity costs were minor in the year following a hip fracture, due to the high age of the population. The first month after injury was the most costly; healthcare costs accumulated to €6932. The majority of costs in the first month were related to hospitalizations (> 50% of total monthly costs). The average length of stay in the hospital following the fracture was 8.6 days (median of 7; range of 1–63). The second largest cost component was long-term care stay (> 40%). Almost 54% of patients returned home after hospital discharge. As time progresses, average monthly costs decreased, from over €3193 in months 2 and 3 after injury to €1206 in months 7 to 12 after injury. In months 7 to 12 after injury, the vast majority of monthly costs (> 80%) were due to long-term care stay.

**Table 2:** Average costs (in euros)

	Total costs			Monthly follow-up costs							
	Total costs	Procedure costs (*n* = 321)	Annual follow-up costs (n = 663) *	Month 1 (*n* = 663) *		Months 2–3 (*n* = 652)		Months 4–6 (*n* = 621)		Months 7–12 (*n* = 598)	
	Mean	Mean	Mean	Mean	Range	Mean	Range	Mean	Range	Mean	Range
Medical costs	27,466	2706	24,760	6932	1005-18,134	3193	0–26,645	1770	0–20,489	1205	0–15,770
Productivity costs	107	N/a	107	61	0–10,125	16	0–2394	2	0–1551	1	0–849
Total	27,573	2706	24,867	6993	1005–18,134	3209	0–26,645	1773	0–20,489	1206	0–15,770

*Excluding procedure costs

### Subgroup analyses

Table 3 presents the results of the subgroup analyses. Although follow-up costs for patients who died within the study period were higher than for patients who survived the first year after injury, the confidence intervals around these estimates were overlapping. Male patients incurred significantly more QALYs than female patients. Younger patients on average had lower follow-up costs and more QALYs than older patients. Costs for patients with comorbidities were higher and health effects than for patients without comorbidities. Confidence intervals of costs for patients living at home pre-injury and institutionalized patients overlapped. QALYs were significantly higher for patients living at home pre-injury. Costs for frail elderly patients were higher than for non-frail patients. QALYs were significantly higher for non-frail patients.

**Table 3:** Subgroup analyses

	Number	Total annual follow-up costs *		QALYs	
		Mean	95% CI	Mean	95% CI
All patients	625	€25,395	€21,569–€29,221	0.528	0.504–0.553
Deceased					
Alive after 12 months	516	€23,909	€19,887–€27,931	0.601	0.578–0.624
Not alive after 12 months	109	€32,431	€22,462–€42,401	0.185	0.138–0.231
Gender					
Male	187	€19,967	€13,960–€25,975	0.588	0.546–0.630
Female	438	€27,712	€22,845–€32,580	0.503	0.473–0.532
Age at injury					
65–69	101	€9911	€3423–€16,399	0.714	0.673–0.754
70–79	177	€14,822	€9341–€20,303	0.634	0.591–0.676
80–89	246	€32,651	€26,203–€39,100	0.457	0.419–0.494
≥ 90	101	€41,733	€28,756–€54,711	0.333	0.277–0.388
Comorbidity					
No comorbidities	108	€16,371	€10,337–€22,405	0.731	0.689–0.772
One comorbidity or more	511	€27,418	€22,882–€34,954	0.485	0.459–0.512
Pre-injury living situation					
Home	486	€26,778	€22,198–€31,357	0.600	0.576–0.625
Institutionalized	132	€20,709	€12,562–€28,856	0.264	0.220–0.308
Frailty pre-injury					
Non-frail (GFI < 4)	296	€16,044	€11,837–€20,250	0.725	0.701–0.749
Frail (GFI ≥ 4)	329	€33,942	€27,623–€40,260	0.348	0.318–0.379

*Excluding procedure costs

## Discussion

This study assessed the burden of illness of hip fractures. Approximately 20% of patients died within 1 year. Patients experienced reduced HRQOL and did not recover to their pre-injury HRQOL level within 1 year. Average annual healthcare costs were €27,573, of which 10% was related to the surgical procedure and 90% was due to follow-up costs in the first year after injury. Follow-up costs were found to be concentrated in the first period after injury.

### Comparison to other studies

The findings on the pattern of HRQOL after a hip fracture in the current study concur with earlier findings in a recent literature review, in that HRQOL is severely reduced after a hip fracture and patients recover to a level below their pre-injury HRQOL [5]. With respect to costs, other studies also found that incremental costs declined over time since injury [9, 23–25]. Comparing international cost estimates is complicated due to international differences in, among others, unit cost prices, inclusion of cost categories, finance systems, healthcare pathways and patient populations.

Over the years, various studies assessed per patient costs of hip fractures in the Netherlands. In 1999, per patient were estimated to be €13,600 (inflation corrected to 2018: €19,741) [7]. A study that collected data between 2003 and 2007, estimated healthcare costs of €18,233 (2018: €21,975) [8]. Another study, with data collected between 2008 and 2011 estimated healthcare costs for the first year to be 23,869 (2018: €26,355) [9]. Using 2012 data, per patient healthcare costs were estimated to be €19,717 (2018: €21,770), with additional costs due to lost productivity of €34,518 (2018: €38,113) [10]. When comparing the results of the current study to previous Dutch studies, the current estimates of healthcare costs resemble findings in three of these earlier studies [8–10]. The study by Meerding et al. estimated lower healthcare costs (€13,600; 2018 values €19,741) for a period of 9 months after injury [7]. Costs were lower than in the current study for multiple reasons: the follow-up period was shorter than in the current study; institutionalized patients were excluded; and lower unit cost prices were used. Productivity costs were previously estimated in one Dutch study [10], and were much higher than estimated in the current study, which was likely to be explained by the high age of the patient population; only elderly patients were included in the current study. As such, the majority of patients was already retired and did not incur productivity costs from paid work.

### Limitations

Cost estimates of the surgical procedure were based on information from one hospital only, supplemented with expert opinion. This hospital is a level 1 trauma centre (i.e. the highest level in the Netherlands), which might not be representative for all hospitals in the Netherlands. Ideally, multiple hospitals with varying levels of trauma care would have been included, and expert input would have been replaced by observed parameter input on involvement of medical personnel. Furthermore, procedure costs were not determined for the same patients who were included in the follow-up cost study in BIOS. Combining the data into a single cost estimate therefore assumes that the procedure costs can be generalized to the patients in the BIOS study.

Besides hospital costs related to the surgical procedure and an assumed one-time follow-up outpatient visit, hospital-related resource use was not measured in the BIOS study. Follow-up hospitalizations due to complications were not included in the study either. This has resulted in an underestimation of total costs. In addition, the dataset did not contain information on informal care use. As such, costs related to informal care could not be taken into account. An earlier study in informal caregivers in a subsample of this patient population showed that the use of informal care is substantial: the vast majority of patients had received informal care (only 11% of contact persons had never provided informal care); in the first month after injury, patients on average received 50 h of informal care per week, and after 6 months, patients received 25 h of informal care per week [6]. Such volumes of care are associated with monthly costs of €2740 and €1370, respectively. Considering the size of total monthly costs calculated in this study (€6933 and €1929 in months 1 and 6, respectively), the absence of informal care costs is therefore an important hiatus of the study. Finally, no data were collected on productivity costs from unpaid work. Because the majority of patients are already retired in this patient population, productivity costs from paid work are limited. Performing unpaid work is less age dependent, e.g. a patient aged 90 might still be able to perform household activities. Therefore, hip fractures potentially lead to productivity losses and associated costs from unpaid work in this patient population. Future research could focus on this type of productivity losses.

Data were only collected from patients who were willing to participate in the BIOS study. Patients that died during the initial hospitalization after the hip fracture were therefore not included in the sample. Likewise, patients with a very bad prognosis might have opted not to participate in the study as well. This selection bias might have led to an underestimation of the burden of illness. This is apparent from the 30-day survival rate in BIOS (99.5%), which is much lower than the mortality in the total hip fracture populations with a 30-day survival rate of 86.7% reported in a systematic literature review [26].

Pre-injury HRQOL was determined retrospectively. This may have caused recall bias. Prospective data collection is not possible for pre-fracture patients. A recent systematic literature review showed that use of retrospective assessment of pre-injury quality of life is the most common method to collect quality of life before injury; this method was used in 29 of 31 identified studies [27]. The use of population values has been suggested as an alternative. However, these might not be an adequate reflection of people with high risk of hip fractures, as these high-risk people might already have more health problems and worse HRQOL compared to matched controls in the overall population, as was indicated by the 13% lower utility values of pre-injury found in this study.

### Implications

The results of this burden of illness study can be used in future economic evaluations in elderly patients with hip fractures. In particular, the distinction of utility values and monthly costs at different points in time after surgery can prove useful for health economic modelling, especially when costs and effects are extrapolated beyond the follow-up period of the study.

The objective of this study was to estimate the burden of illness in elderly Dutch patients. Hence, we adhered to Dutch guidelines with respect to quantifying utilities and costs. The use of utility tariffs for other countries might result in different utility values, but the pattern of HRQOL over time after injury is unlikely to be different in other countries. This study used the friction cost method to monetarize productivity losses from paid work. Alternatively, the human capital method could have been used. However, the results would be similar, since the majority of patients in the sample were over the retirement age.

## Conclusions

Patients with hip fractures experience a significant burden in the period after injury, as they experience an increased mortality risk and reduced HRQOL. In addition, hip fractures lead to a substantial economic burden, particularly due to costs of healthcare consumption. The results of this study can be used in future cost-effectiveness studies.

## Electronic supplementary material

ESM 1 (PDF 161 kb)
