# Supplementary material for: Burden of illness of hip fractures in elderly Dutch patients
Source: Arch Osteoporos. 2020 Jan 3;15(1):11. doi: 10.1007/s11657-019-0678-y (PMC6940317; doi:10.1007/s11657-019-0678-y)
Supplement: Supplementary file 1 — (PDF 161 kb) [file 11657_2019_678_MOESM1_ESM.pdf]

Missing data

Health-related quality of life

To ensure the optimal use of available data, missing data were imputed in a multi-step process. Firstly, individual missing dimensions of the EQ-5D were imputed using multiple imputation, using chained equations to enable imputation of missing values in multiple variables [1]. The non-missing dimensions of the EQ-5D and VAS at the same time point, plus gender and age at injury were used as independent variables. Secondly, if T2 EQ-5D questionnaires were missing in its entirety, the EQ-5D dimensions at T2 were imputed using multiple imputation, using all five EQ-5D dimensions at T1, the same EQ-5D dimension at later time points, gender and age at injury as independent variables. Thirdly, when EQ-5D data were missing in its entirety at a particular measurement other than T2, linear interpolation between time points of utility values was applied. Linear interpolation was only applied for time points after surgery. When utility scores at T5 were missing, these were assumed equal to utility scores at T4. Finally, EQ-5D utilities were imputed using multiple imputation, using all available EQ-5D utilities at other time points as independent variables. Table A1 shows the percentages of missing items that were imputed at each step of the imputation procedures.

Imputation of EQ-5D dimensions (step 1 and 2) were performed using ordered logit regression models, because EQ-5D dimensions are categorical variables. For imputations of utility values (step 4) predictive mean matching was used [2]. Predictive mean matching provides a linear prediction and imputes missing values by nearest-neighbour donor, with the distance based on the predicted value of the missing variable from the linear regression. Using predictive mean matching ensures that the distributions of utility values (bound between -0.329 and 1.000 in the Dutch value set) are preserved.

Table A1 Details imputation procedure EQ-5D dimensions and utilities

Imputed variable	Complete items	Missing items	Total items	% missing
Step 1. Imputing single EQ-5D Dimensions using multiple imputation				
T0 EQ-5D Mobility	651	5	656	0.8%

T0 EQ-5D Self care	652	4	656	0.6%
T0 EQ-5D Usual activities	650	6	656	0.9%
T0 EQ-5D Pain / Discomfort	644	12	656	1.8%
T0 EQ-5D Anxiety / Depression	652	4	656	0.6%
T1 EQ-5D Mobility	232	7	239	2.9%
T1 EQ-5D Self care	239	0	239	0.0%
T1 EQ-5D Usual activities	238	1	239	0.4%
T1 EQ-5D Pain / Discomfort	239	0	239	0.0%
T1 EQ-5D Anxiety / Depression	238	1	239	0.4%
T2 EQ-5D Mobility	591	10	601	1.7%
T2 EQ-5D Self care	597	4	601	0.7%
T2 EQ-5D Usual activities	595	6	601	1.0%
T2 EQ-5D Pain / Discomfort	595	6	601	1.0%
T2 EQ-5D Anxiety / Depression	597	4	601	0.7%
T3 EQ-5D Mobility	618	4	622	0.6%
T3 EQ-5D Self care	620	2	622	0.3%
T3 EQ-5D Usual activities	616	6	622	1.0%
T3 EQ-5D Pain / Discomfort	617	5	622	0.8%
T3 EQ-5D Anxiety / Depression	618	4	622	0.6%
T4 EQ-5D Mobility	551	7	558	1.3%
T4 EQ-5D Self care	558	0	558	0.0%
T4 EQ-5D Usual activities	557	1	558	0.2%
T4 EQ-5D Pain / Discomfort	550	8	558	1.4%
T4 EQ-5D Anxiety / Depression	557	1	558	0.2%
T5 EQ-5D Mobility	540	4	544	0.7%
T5 EQ-5D Self care	544	0	544	0.0%
T5 EQ-5D Usual activities	542	2	544	0.4%
T5 EQ-5D Pain / Discomfort	537	7	544	1.3%
T5 EQ-5D Anxiety / Depression	540	4	544	0.7%
Step 2. Imputing single T2 EQ-5D dimensions using multiple imputation				
T2 EQ-5D Mobility	591	17	608	2.8%
T2 EQ-5D Self care	597	16	613	2.6%
T2 EQ-5D Usual activities	595	17	612	2.8%
T2 EQ-5D Pain / Discomfort	595	16	611	2.6%
T2 EQ-5D Anxiety / Depression	597	16	613	2.6%
Step 3. Imputing utility values using linear interpolation				
T2 Utility value	9,170	416	9,586*	4.3%
T3 Utility value	8,783	1148	9,931*	11.6%
T4 Utility value	7,761	1150	8,911*	12.9%
T5 Utility value	8,423	264	8,687*	3.0%
Step 4. Imputing utility values using multiple imputation				
T1 Utility value	230	411	641	64.1%
T2 Utility value	621	52	673	7.7%
T3 Utility value	674	112	786	14.2%
T4 Utility value	656	121	777	15.6%
T5 Utility value	695	95	790	12.0%

* Imputed dataset

Costs

Missing resource items in completed iMCQ questionnaires were imputed using the mean value for each individual item. Multiple imputation was not deemed appropriate for resource use items due to volatility and unpredictability of resource use. If iMCQ questionnaires were missing in its entirety at a particular measurement, total costs were imputed using multiple imputation, using age at injury, health-related quality of life and costs at all available time points as independent variables. Before the multiple imputation procedure, costs were log-transformed to account for non-normality in the data. Table A2shows the percentages of missing items of costs that were imputed in the imputation procedure.

Table A2 Details imputation procedure healthcare costs

Imputed variable	Missing items	Total	% missing
T2 Total healthcare costs	125	671	18.6%
T3 Total healthcare costs	218	793	27.5%
T4 Total healthcare costs	284	748	38.0%
T5 Total healthcare costs	318	750	42.4%

References

1. White IR, Royston P, Wood AM (2011) Multiple imputation using chained equations: issues and guidance for practice. Stat Med 30:377-99

2. Little RJA (1988) Missing-data adjustments in large surveys. J Bus Econ Stat 6:287-96
